# Path Configuration Complexity Affects Spatial Memory Span on the eCorsi Task but Does Not Influence Performance of a Concurrent Auditory Discrimination Task

**DOI:** 10.3390/vision7010024

**Published:** 2023-03-18

**Authors:** Anthony Tapper, Ewa Niechwiej-Szwedo

**Affiliations:** Department of Kinesiology and Health Science, Faculty of Health, University of Waterloo, Waterloo, ON N2L 3G1, Canada; eniechwi@uwaterloo.ca

**Keywords:** divided attention, working memory, Corsi, spatial memory

## Abstract

Visuospatial working memory is often assessed using the Corsi block-tapping task where set size is used to estimate capacity. It is well established that characteristics of the Corsi task path configuration such as length, crossings, and angles influence recall accuracy suggesting that more complex path configurations increase the load on working memory. However, the interaction between set size and path configuration is not well understood. Here we used a secondary auditory task to probe if set size and path configuration impose a similar type of load on the system. Nineteen participants (age = 25.3 ± 3.9 years) performed a computerized version of the Corsi test either alone (single) or simultaneously with an auditory tone discrimination task (dual). The eCorsi task involved a set of simple (no crosses, shorter lengths, larger angles) or complex (>2 crosses, longer lengths, smaller angles) paths at set sizes of five to eight blocks. Results showed significantly lower recall accuracy for the complex compared to the simple paths (63.32% vs. 86.38%, *p* < 0.001) at all set sizes, regardless of task condition (single, dual). Auditory performance (accuracy and response time) was significantly lower in the dual compared to single task (85.34% vs. 99.67%, *p* < 0.001), but performance was not affected by the complexity of the eCorsi path configuration. These findings suggest that set size and path complexity impose a different type of load on the working memory system and may rely on different resources.

## 1. Introduction

Working memory refers to the limited amount of information that can be temporarily held for use in everyday tasks [1], for instance, a set of written or verbal instructions to cook our favorite meal or remembering someone’s email address. Working memory capacity is commonly assessed using tasks that progressively vary the number of items that need to be recalled [1,2]. The Corsi block-tapping task (CBTT) is often used to evaluate visuospatial working memory capacity which is defined as the highest number of items successfully recalled in serial order [3,4]. The traditional CBTT requires a participant to encode and recall a set of block locations that were touched in some order by an experimenter. In the computerized version, the blocks change color or illuminate in serial order. The most common manipulation of working memory load in the CBTT is progressively increasing the number of blocks to recall (i.e., set size). Importantly, altering the path characteristics within a set size has a significant influence on recall performance suggesting that the difficulty of a spatial working memory task is not solely dependent on the number of items (i.e., set size) [5,6,7,8,9,10,11]. Hence, the estimate of working memory capacity obtained using the CBTT task is influenced by set size and the complexity of a path configuration (i.e., crosses, angles, and distance). However, the interaction between set size and path complexity is not well understood and it remains to be determined whether set size and path complexity represent a similar type of load on the working memory system. Lavie’s load theory could provide some insight into this question. Specifically, the theory makes a distinction between perceptual and cognitive load, which have a differential effect on an individual’s ability to ignore irrelevant distractor stimuli. In the present study, performance on a secondary auditory discrimination task was assessed as participants responded to a relevant target while ignoring an irrelevant stimulus. Thus, the auditory task was used to probe the type of load imposed by set size and path configuration complexity. It is important to understand the type of load imposed by changing task characteristics because this can help in the development of a valid and reliable test for assessing neurocognitive processing in individuals with neurodegenerative diseases or those who have suffered a mild traumatic brain injury (e.g., concussion).

Load theory makes a distinction between perceptual and cognitive load. Perceptual load is defined as the amount of information that can be processed in a limited attentional capacity system and it depends on external task-related properties [12,13]. It is commonly studied by instructing participants to perform a primary visual search task or to encode blocks presented in a single display, while ignoring a peripheral distractor stimulus (e.g., superimposed human face or small shape), which is followed by a probe to assess whether the peripheral distractor was processed or not. Research has shown that a higher perceptual load induced by increasing the number of items to be encoded [12,14,15], increasing the number of incongruent flankers in a visual search task [16], or reducing the discriminability between an object’s features (i.e., size and/or shape) compared to another object [17], is associated with reduced processing of an irrelevant distractor [13]. For example, low perceptual loads are associated with higher accuracy and shorter response times on a primary task and greater sensitivity in detecting a distractor. In contrast, high perceptual loads cause longer response times and higher error rates on the primary task and reduced distractor detection [13]. It has been proposed that distractors are more difficult to ignore when the perceptual load of the primary task is low because ‘spare’ attentional capacity is available (or ‘spills-over’) to process a distractor. In contrast, when perceptual load of the primary task is high, sensory processing capacity becomes bottlenecked such that only relevant information is perceptually processed (i.e., primary task) whereas irrelevant distractors are not processed due to limits in the perceptual capacity [18]. This process is thought to be indicative of more focused attention and early selection.

Cognitive load, referred to as “executive control load” reflects an individual’s inherent abilities such as working memory capacity [18]. It is often studied using a “sandwich” task where participants maintain a set of verbal items in working memory while ignoring a visual, verbal, or tactile distractor or while simultaneously performing a response-competition task (e.g., responding as quickly as possible to a target letter in the presence of a congruent or incongruent distractors) [12,15,19,20]. The main manipulation of cognitive load involves increasing the number of items to *maintain* in working memory (e.g., increasing Corsi set size) or changing the order of items to be *maintained* (e.g., six digits in ascending order “low load” versus random order “high load”) [12,13,15,20]. Studies have reported that increasing cognitive load has an opposite effect on distractor processing compared to perceptual load (i.e., increased sensitivity in detecting a peripheral distractor when cognitive load is high) [12,15,20]. These results are thought to be indicative of less focused attention under higher cognitive loads or an impaired prioritization process where distractors compete for processing resources [13]. An influential study [12] found that increasing the number of colored blocks to be encoded or maintained in working memory reduced visual distractor processing whereas increasing the number of verbal items (i.e., letters) to be maintained increased visual distractor processing. This finding suggests that encoding and maintaining items in working memory increases distractor rejection if the tasks share the same perceptual resources (i.e., visuospatial), as reflected in perceptual load; in contrast, when the working memory task involves different resources (i.e., verbal and visuospatial), distractor processing is increased, as reflected in cognitive load. To summarize, the general principle of load theory is whether there is a limitation in the availability of attentional resources (i.e., perceptual load) or a limitation in priority-based control of attentional resources (i.e., cognitive load).

The Corsi task has been used in previous research to test the components of Baddeley’s WM model [2,21,22]. In general, performing a secondary auditory tone discrimination task simultaneously during the encoding phase is associated with poorer auditory task performance but no difference in recall performance compared to when performing each task alone [23]. These findings are consistent with Baddeley’s framework of working memory [24,25,26,27,28] suggesting that the Corsi task requires support from the visuospatial sketchpad and central executive systems. Previous studies have two main limitations: (1) they did not control for path configuration characteristics within a set size [27,28], which has been shown to influence encoding and recall [5,6,7,8,9,10,11]; or (2) they did not provide specific instruction on which task to prioritize in the dual-task condition [24,25,26]. Thus, the question remains whether the load imposed by set size and path complexity reflects cognitive or perceptual load.

Previous research offers some insight into this question. For instance, Bor [5] found greater activation in the dorsolateral prefrontal cortex (DLPFC) and better recall performance when participants encoded symmetrical Corsi sequences (i.e., right angled and parallelograms) compared to unsymmetrical sequences (i.e., more complex path with crosses and smaller angles). The authors suggested that the increased DLPFC activity facilitated memory encoding by efficiently chunking information together resulting in a reduced cognitive load. On the other hand, path complexity may impose a greater load on perceptual processing capacity because perceptually grouping items to conform to the Gestalt principle (i.e., similarity, proximity, continuity, etc.; see review [29]) is dependent on selective attention. Specifically, sequences that are more symmetrical (i.e., follow smooth and continuous trajectories) require less attention for perceptual grouping compared to asymmetrical sequences. Thus, if path complexity affects perceptual load, we would expect reduced overall performance on the secondary auditory task when encoding more difficult sequences because more attention will be required to perceptually process the difficult sequences leaving less resources available for the auditory task. Specifically, according to Lavie’s model, higher perceptual load should be associated with fewer errors of commission. Alternatively, if path configuration complexity imposes a cognitive load, we would expect more errors of commission.

To summarize, the goal of this study was to investigate the type of load imposed by eCBTT path complexity using a secondary auditory oddball task. It was hypothesized that increasing path complexity within a set size will increase perceptual load which will be associated with longer response times, lower oddball accuracy, and decreased errors of commission. A secondary goal of this study was to assess the effects of load on performance in individuals with a history of concussion. Notably, the current study might offer insight into what type of load (perceptual or cognitive) is most sensitive to the chronic effects of concussion.

## 2. Materials and Methods

Participants. Nineteen university students participated in the study (see Table 1 for cohort demographics). Visual acuity was assessed using a Bailey–Lovie chart. Participants were excluded if they did not have normal or corrected-to-normal acuity, reported a medical history of neurological disorders, cardiovascular disease, or were currently taking medication affecting the central nervous system. Participants completed a modified version of the Waterloo health history questionnaire which asked questions regarding symptoms related to concussion. Ten participants reported having a previous medically diagnosed concussion (>1-year post-injury) but had recovered and returned to regular daily activities and were asymptomatic at the time of testing.

**Figure 1:**
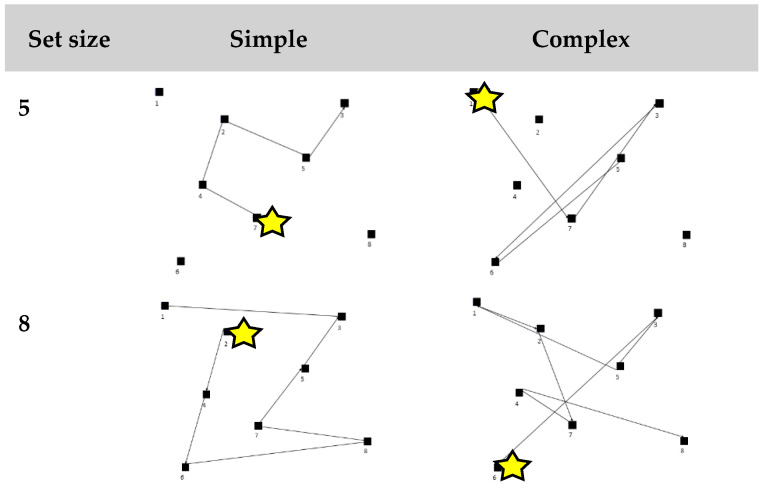
Example of a simple and complex path at set sizes of five and eight blocks. The yellow star indicates the first block presented in the path and the lines are connected in sequential order of block presentation.

eCorsi block task. The eCorsi was created using VPixx 3.2.1 software (VPixx Technology Inc., Montarville, Canada) and administered on a Macbook Pro laptop (OS X, version 10.8.5). Participants sat comfortably 60 cm away from the screen and were free to make natural head and eye movements throughout the task. Eight black blocks were displayed on a grey background. The blocks changed color from black to white every 750 ms in a specific sequence. Participants were instructed to remember the sequence of blocks in the same order in which it appeared. The test began with a sample trial performed by the investigator at a set size of three, followed by a practice trial performed by the participant. Next, four practice sequences at a set size of four blocks were presented to familiarize participants with the progressive increase in set size. The testing condition involved six sequences presented at each set size increasing from five to eight blocks. Each set size had three trials that were categorized as “simple” which was defined as sequences with no path crossings, an average angle greater than 70 degrees, and “shorter” distances between target blocks presented (range: 255–685 mm). The other three sequences were categorized as “complex” defined as sequences with more than two path crossings, an average angle less than 50 degrees, and “longer” path distances between targets presented (range: 530–945 mm). Distances between blocks in the more complex sequences were 10–60% longer compared to the simple sequences with the same set size (see Figure 1). Simple and complex sequences were randomized within a set size.

Auditory oddball task. Auditory tone pips (5 ms duration, with 1 ms rise/fall time) were created using VPixx 3.2.1 software and presented binaurally at 60 decibels using a Macbook Pro laptop (OS X, version 10.8.5). First, participants were presented with an iteration of high (1000 Hz) and low (325 Hz) tones and asked if they could discriminate between the two tones. All participants responded “Yes”, confirming they could correctly discriminate the two tones. Then, participants were instructed to respond by clicking a computer mouse with their preferred hand as quickly as possible when a high tone was presented (*probability* = 40%), and to withhold a response when a low tone was presented (*probability =* 60%). The testing protocol began with the participant clicking the central fixation followed by an iteration of high and low tones presented at a fixed interstimulus interval of 700 ms. Participants were presented with a sample trial performed by the investigator involving a five-tone pip iteration (equivalent to set size of three in the eCorsi task) prior to performing the practice trial. Four tone iterations at set size four (i.e., six tones) were used to further familiarize participants with the task. The testing condition involved six trials presented at set size five (seven tones) to eight (ten tones). In every trial, target tones were separated by at least one non-target tone. Of the 204 tones presented throughout the single-task condition, 84 were high tones (target tone) and 120 were low tones (non-target tone). The same three tone iterations presented in the simple sequence condition were presented in the complex sequence condition within the same set size.

Dual task. The third testing condition involved performing both eCorsi block and auditory oddball tasks simultaneously. Participants were first shown a practice trial performed by the investigator at a set size of three (five auditory tones). Then, participants performed four practice trials at a set size of four (six auditory tones). The trial began by clicking on the central fixation followed by auditory tones presented every 700 ms and the eCorsi blocks changing color every 750 ms. Participants were asked to respond to a high tone and withhold a response to a low tone while encoding the eCorsi sequence. Instructions were given to prioritize the eCorsi test in the dual-task condition.

## 3. Results

### 3.1. Preliminary Analyses

The cohort tested in the study included participants with a history of concussion; although, they were asymptomatic at the time of testing, research shows that these individuals have residual difficulties in information processing that may affect their performance on the dual task [30]. Thus, prior to conducting the main analysis which aimed to assess the effect of path complexity on the performance of the secondary auditory oddball task, omnibus analyses with concussion history included as a between subject factor were performed on each outcome measure to determine any differences in the pattern of performance between the groups.

eCorsi task. To assess whether participants followed instructions by prioritizing the eCorsi in the dual-task condition, a paired-test was performed on eCorsi recall accuracy between task conditions (single, dual). Results showed no significant difference between task conditions *t*(18) = 1.66, *p* = 0.115 (no concussion: *t*(8) = 1.92, *p* = 0.091; history of concussion: *t*(9) = 0.65, *p* = 0.530), indicating all participants followed instructions.

**Figure 2:**
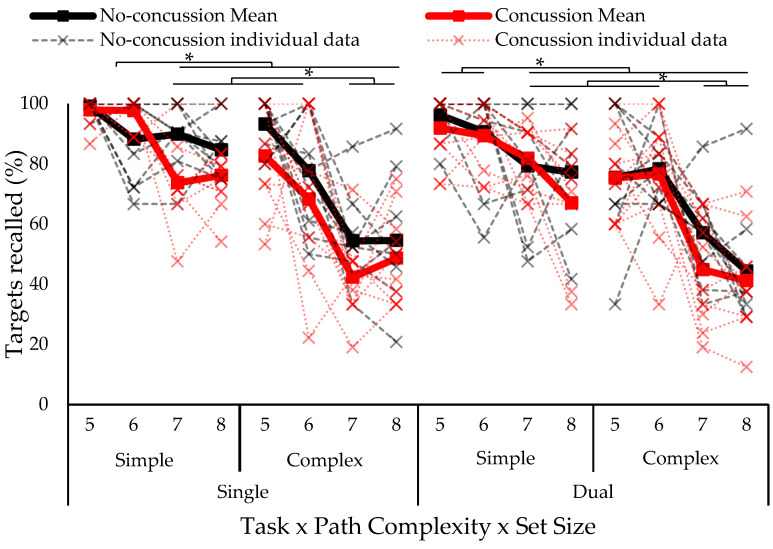
eCorsi recall accuracy separated by group (concussion, no concussion), path complexity (simple, complex), and set size (five to eight blocks). Solid lines indicate group means and individual data is represented in dotted lines. Asterisk indicates statistically significant difference; *p* < 0.05.

The omnibus four-way ANOVA test on eCorsi recall accuracy revealed a significant three-way concussion history by path complexity by set size interaction (*F*_6,51_ = 3.15, *p* = 0.011, *ƞ*^2^ = 0.029). Tukey–Kramer post hoc showed lower accuracy for the complex compared to simple paths at all set sizes in both groups (no concussion, history of concussion). The group with a history of concussion had decreased accuracy at set sizes of seven and eight compared to five and six (five and six blocks > seven and eight blocks). The no-concussion group had a similar recall accuracy at a set size of six compared to sizes of seven and eight for the simple paths (see Figure 2).

Auditory oddball task. To assess the effects of dual-tasking on auditory oddball performance, three separate paired-tests were performed on outcome variables auditory oddball accuracy (% of target tones correctly responded to), errors of commission (% of non-target tones incorrectly responded to), and response time (ms; to target tones). Results showed significantly lower accuracy in the dual-task condition compared to the single-task *t*(18) = 5.11, *p* < 0.001 (no concussion: *t*(8) = 6.77, *p* < 0.001; history of concussion: *t*(9) = 11.14, *p* < 0.001), higher errors of commission *t*(18) = 4.41, *p* < 0.001 (no concussion: *t*(8) = 4.20, *p* = 0.003; history of concussion: *t*(9) = 4.33, *p* = 0.002), and longer response times *t*(18) = 9.05, *p* < 0.001 (no concussion: *t*(8) = 5.59, *p* = 0.005; history of concussion: *t*(9) = 7.31, *p* < 0.001). This further validated that the auditory task was the secondary task in the dual condition.

**Figure 3:**
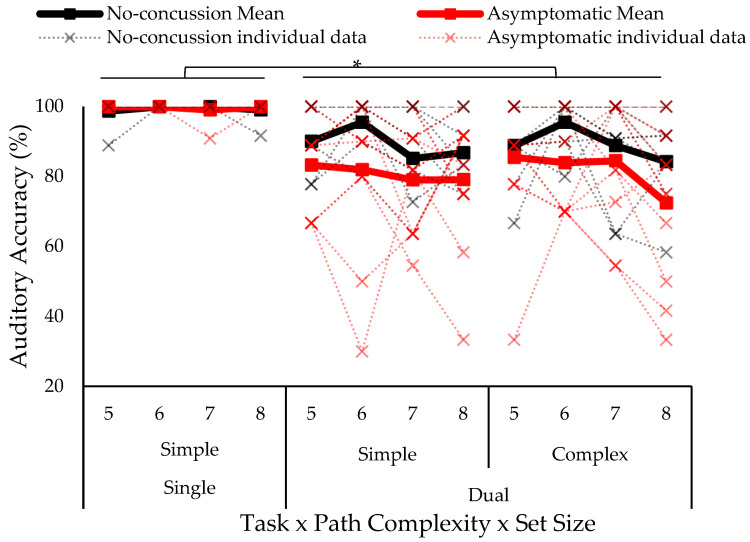
Auditory accuracy separated by group (no concussion, history of concussion), task (single, dual), path complexity (simple, complex), and set size (five to eight blocks). Solid lines indicate group means and individual data is represented in dotted lines. Asterisk indicates statistically significant difference; *p* < 0.05.

The omnibus four-way ANOVA test on auditory accuracy (% of tones correctly responded to) showed a significant three-way concussion history by task condition by set size interaction (*F*_6,51_ = 2.57 *p* = 0.029, *ƞ*^2^ = 0.015) (see Figure 3). Tukey–Kramer post hoc revealed that both groups had lower accuracy in the dual-task compared to single-task; moreover, those with a history of concussion had lower accuracy at all set sizes in the dual-task condition compared to the no concussion group (no concussion: 89.4% ± 10.8; concussion history: 81.2% ± 19.2); thus, replicating previous findings [23,30]. No significant between-group differences were shown in the single task.

For auditory errors of commission, there was a significant interaction of concussion history and task (*F*_1,17_ = 4.60 *p* = 0.047, *ƞ*^2^ = 0.026). Post hoc revealed a greater number of errors in the group with a history of concussion (3.4% vs. 1.7%) in the dual task (Table 2). No difference was found in the single task (both groups < 0.5% error).

**Figure 4:**
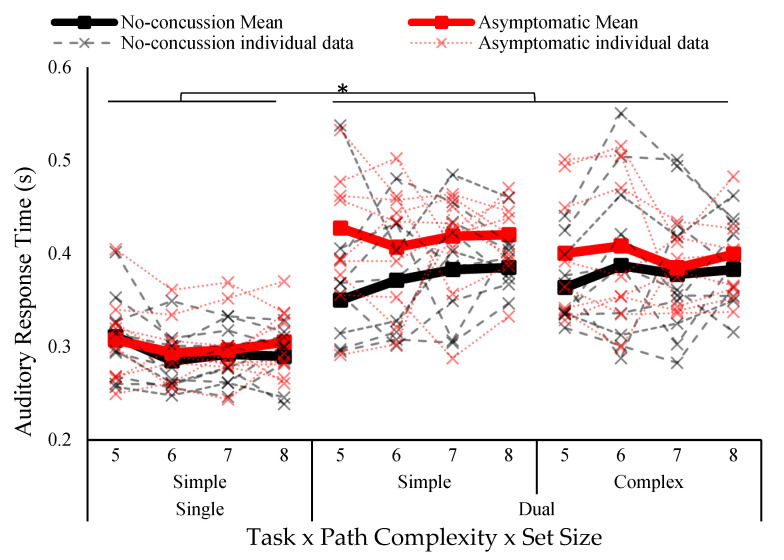
Auditory response time separated by group (no concussion, concussion history), task (single, dual), path complexity (simple, complex), and set size (five to eight blocks). Solid lines indicate group means and individual data is represented in dotted lines. Asterisk indicates statistically significant difference; *p* < 0.05.

Auditory oddball response time is illustrated in Figure 4. There was a main effect of task (*F*_1,17_ = 80.74 *p* < 0.001, *ƞ*^2^ = 0.494). Response time was shorter in the single task (295 ± 33 ms) compared to the dual task (393 ± 60 ms). No other effects reached significance.

### 3.2. Effects of Path Complexity on Auditory Oddball Task Performance

To test the main hypothesis assessing how the type of load imposed by eCorsi path complexity affected auditory oddball performance, a three-way ANOVA was performed on auditory oddball accuracy (% of target tones correctly responded to), errors of commission (% of non-target tones incorrectly responded to), and response time (to target tones). History of concussion was included as a between-subject variable, and the two within-subject variables included path complexity (simple, complex) and set size (five to eight blocks). Tukey–Kramer post hoc test was used to compare differences between the means when the main effects or interactions reached significance.

**Figure 5:**
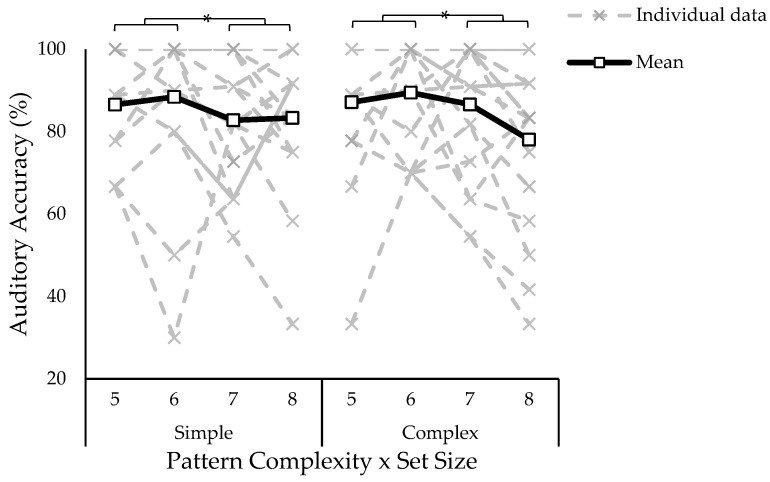
Auditory accuracy represented as the percentage of target tones correctly responded to in the dual-task condition. The single task was not plotted because it was performed with 100% accuracy. The *x*-axis is separated by path complexity (simple, complex) and set size (five to eight blocks). There were no differences in accuracy due to path complexity, but accuracy decreased as a function of set size (five and six > eight). Asterisk indicates statistically significant difference; *p* < 0.05.

A three-way ANOVA on auditory accuracy in the dual-task condition revealed no significant effect of path complexity (*F*_1,17_ = 0.20, *p* = 0.657, *ƞ*^2^ = 0.000), and no path complexity by set size interaction (*F*_3,51_ = 0.94, *p* = 0.428, *ƞ*^2^ = 0.010). There was a significant main effect of set size (*F*_3,51_ = 4.48, *p* = 0.007, *ƞ*^2^ = 0.035). Tukey–Kramer post hoc showed reduced auditory accuracy when presented with the largest set size (i.e., eight blocks) compared to set sizes of five and six blocks (see Figure 5).

A three-way ANOVA performed on auditory errors of commission and response time in the dual-task condition revealed no significant main effect of path configuration or interactions (*p* > 0.22 for all effects). As shown in Table 2, errors of commission were higher in the group with a history of concussion but there was no effect of path complexity.

## 4. Discussion

The main goal of the study was to examine the type of load imposed by the eCorsi path complexity. Our manipulation of eCorsi complexity (i.e., crosses, angles, distance) within a set size was successful because recall accuracy was lower for the complex paths compared to the simple paths at all set sizes. Notably, the complex paths at lower set sizes (five and six blocks) were recalled with similar accuracy as the simple paths at higher set sizes (seven and eight blocks). Contrary to our hypothesis, eCorsi path complexity did not affect the auditory oddball task performance indicating that it is unlikely that eCorsi path complexity influences perceptual load. Consistent with our previous work, auditory oddball performance was affected by concussion history [23] indicating that those with a history of concussion have difficulty when performing two tasks simultaneously. Thus, individuals with a history of mild traumatic brain injury (mTBI) might have a reduced availability of attentional capacity compared to those without a TBI.

Although our dual-task paradigm was not ideally suited to directly test Lavie’s theory because the secondary task was not a pure “distractor” task, the theory still provides a useful framework to assess the type of load imposed by path configuration complexity. Our secondary task was not a pure “distractor task” because the auditory information must have been attended to some extent in order to make a decision whether the tone is a target and respond, or to withhold a response if the tone was not a target. Previous studies testing Lavie’s theory employed a pure distractor task where the information was not relevant at all. Our findings do not support the idea that eCorsi path complexity imposes a perceptual load. If perceptual load was increased, we would have expected errors of commission to decrease; however, the results did not reveal any significant effects for errors of commission due to path complexity. Instead, results are consistent with previous research by Bor et al. [5] showing that path complexity reflects some type of executive load. These authors showed that symmetrical paths can be selected into organizational chunks by relating familiar shapes to object-based information held in long-term memory. Interestingly, they found greater activation in the DLPFC (an area associated with attention and executive control), inferior parietal lobule, and fusiform gyrus during encoding of symmetrical compared to asymmetrical paths suggesting that the chunking process might require executive control and consume attentional capacity. Further research by Bor and Seth [31] indicated that attention and memory chunking appear to be dissociable processes that are both supported by an overlapping neural network. Lastly, it is important to acknowledge that our task may impose a combination of perceptual and cognitive load during memory encoding of the eCorsi task because this phase involves both perceptual processing of new items while updating and maintaining older items in working memory.

A large body of research shows reduced performance on the eCorsi task during concurrent performance of a suppression task that relies on the visuospatial sketchpad or the central executive but not the phonological loop [24,25,26,28,32]. For example, Kemps [24] showed a reduction in eCorsi task accuracy as set size increased and this reduction was steeper for asymmetrical (i.e., complex) compared to symmetrical (i.e., simple) paths at set sizes of five to eight blocks; however, when performed simultaneously with a visuospatial suppression task (i.e., matrix tapping task), recall accuracy was reduced to the same degree for both symmetrical and asymmetrical paths. Similarly, other researchers [25,26] found the same degree of reduction in eCorsi accuracy on symmetrical and asymmetrical paths when concurrently performing a central executive suppression task (i.e., verbal trails task) whereas no differences were found for a phonological suppression task (i.e., continuous word repetition). Our findings are consistent with this body of research. Importantly, we instructed participants to primarily focus on the eCorsi task in the dual-task condition which revealed deficits on the auditory task that were unaffected by the complexity of the eCorsi path. In contrast, other studies [24,25,26] instructed participants to perform both tasks together without explicitly instructing which task to prioritize; thus, individuals might have engaged in a strategy where they focused on the suppression task resulting in deficits on the Corsi task. Our study provides some evidence supporting that better recall accuracy of a simple compared to complex path likely reflects a less attentionally demanding process where paths can be reconstructed from common representations held in long-term memory [24,25,26]. In the context of Baddeley’s model, the eCorsi task engages the visuospatial sketchpad and the auditory task relies on the phonological loop, thus, there should be little interference between the tasks. Notably, Baddeley’s model of separate working memory systems has been questioned and not all studies support the specialized storage systems [33,34]. In line with this, our results showed reduced accuracy on the auditory task when it was performed concurrently with the eCorsi task, which indicates some interference. Importantly, the level of interference was not affected by path complexity. In other words, encoding more complex compared to simple paths had no additional effects on auditory performance: there was no significant effect on accuracy, errors of commission or response times.

Our results suggest that path complexity did not impose a perceptual load but likely reflects a less efficient chunking process where complex path configurations do not benefit from common representations held in long-term memory (i.e., redundant shapes). An alternative perspective to the Baddeley’s model on the organization of working memory system was offered by Ruchkin et al. [35]. They suggested that working (short) memory and long-term memory rely on the same neural mechanisms rather than separate storage systems for different types of information. According to this perspective, access to information stored in the posterior cortical regions is mediated by the prefrontal cortex, thus, the limitations on working memory are imposed by activation of the prefrontal cortex, which provides the mechanism to maintain and recall information, as well as prioritize information processing. This interpretation is also consistent with the theory of constructive operators where the M operator represents mental attention (i.e., a limited central resource) and the F operator represents the process of chunking [36]. In the context of our study, the F operator facilitates processing of the simple paths thus reducing the load on the M operator. When set size and path complexity increase, the M operator is fully engaged, most likely exceeding the system’s capacity, thus, recall of the eCorsi sequence is reduced. When the M operator is loaded by the primary task, there is insufficient capacity to perform even a simple auditory discrimination task evinced by decrements in auditory accuracy and response time. Notably, set size had a minimal effect and path complexity had no significant effect on auditory task performance suggesting different mechanisms are involved in supporting the behavior.

Research into the chronic effects of concussion has shown that individuals with a history of concussion perform similarly to a control group on tasks involving working memory [37,38,39,40,41,42] when these tasks are performed alone. Our results are consistent with these studies showing no between-group differences on either task when it was performed alone. In contrast, compared to a control group, individuals with a history of concussion showed reduced performance on the auditory oddball task when performed concurrently with the eCorsi task [23,30]. These deficits might be due to limitations with effective and efficient gating of sensory information when load demands increase [30]. In the context of Lavie’s theory, our findings indicate that tasks loading perceptual processing are helpful in discriminating between individuals with a history of concussion compared to those without. Only one study to our knowledge has examined perceptual load in individuals with a history of mild-to-moderate TBI, ranging from three months to twenty years post injury [42]. They found that individuals with a history of TBI had more errors when detecting a peripheral distractor (i.e., ignored the distractor more often) under low and high perceptual loads compared to a control group. Interestingly, these between-group differences were evident on two separate perceptual load tasks including one designed to replicate Lavie’s original experiment [18] and the other designed to be more ecologically valid by using real-life pictures such as a cup. These results indicate that the availability of attentional capacity for perceptual processing is reduced in individuals with a history of TBI. However, one issue raised by Waters [43] is that participants in the TBI group could have had difficulty remembering task instructions, which might have increased the cognitive load resulting in less efficient allocation of attention. This limitation along with the difficulty to definitively dissociate between perceptual and cognitive load in the encoding phase of our task limits our conclusion about the type of load that is most sensitive in discriminating between groups.

Our study involves some limitations that should be addressed in future research. First, the number of auditory tones presented at each set size was relatively small (i.e., 10–13 target tones per path difficulty per set size). As a result, our estimate of auditory target accuracy and response time in the dual-task condition were based on a relatively small number of tones. Second, the sample size for a between-group comparison was somewhat small (9 controls, 10 asymptomatic) path. Nevertheless, current results replicated previous findings [23,38]. Lastly, our task was not ideally suited to directly test Lavie’s theory, instead the framework was used to probe our understanding of the dual-task paradigm. Future research could test what type of load is most sensitive to concussion history using tasks specifically developed to study load theory [12,15,16].

## 5. Conclusions

The current study examined task-related characteristics that influence performance of the CBTT using a dual-task paradigm. Our results suggest that it is unlikely that path complexity influences perceptual load because performance on the secondary auditory oddball task was not affected by path configuration complexity. Thus, encoding simple paths into working memory might represent a process where common path shapes are integrated with pre-existing representations held in long-term memory resulting in improved recall accuracy compared to complex paths. Importantly, dual-task paradigms appear to be sensitive in discriminating between individuals with a history of concussion. Further investigation into the type of load most sensitive to the chronic effects of concussion will help in the development of a task that can be used by clinicians to help with concussion diagnoses and management.

## Figures and Tables

**Table 1:** Participant Demographics.

Concussion History	Sex	N	Age	Sport Experience	# of Concussions	Symptoms
Control	Female	4	22.20 ± 2.44	13.54 ± 3.44	-	3.5 ± 4.4
	Male	5	26.20 ± 4.65	18.20 ± 4.65	-	3.6 ± 2.8
Asymptomatic	Female	5	24.75 ± 4.27	13.75 ± 1.89	2.4 ± 1.7	7.6 ± 10.5
	Male	5	28.20 ± 4.21	19.20 ± 4.96	1.8 ± 1.1	4.4 ± 6.2

**Table 2:** Mean (%) errors of commission across set size and path complexity in the dual task.

	Set Size 5		Set Size 6		Set Size 7		Set Size 8	
	Simple	Complex	Simple	Complex	Simple	Complex	Simple	Complex
No concussion	1.6 ± 2.4	1.6 ± 2.4	1.0 ± 1.8	0.4 ± 1.3	2.1 ± 1.9	2.9 ± 3.6	2.2 ± 3.7	1.8 ± 3.8
Concussion	3.3 ± 3.2	2.4 ± 3.4	3.7 ± 5.4	2.9 ± 4.4	3.0 ± 2.9	3.0 ± 4.2	5.0 ± 3.9	4.0 ± 4.1

## Data Availability

The data presented in this study are available on request from the corresponding author.

## References

[1] Cowan N. (2016). Working Memory Capacity: Classic Edition.

[2] Redick T.S., Broadway J.M., Meier M.E., Kuriakose P.S., Unsworth N., Kane M.J., Engle R.W. (2012). Measuring working memory capacity with automated complex span tasks. Eur. J. Psychol. Assess..

[3] Berch D.B., Krikorian R., Huha E.M. (1998). The Corsi block-tapping task: Methodological and theoretical considerations. Brain Cogn..

[4] Corsi P.M. (1973). Human Memory and the Medial Temporal Region of the Brain. Ph.D. Thesis.

[5] Bor D., Duncan J., Wiseman R.J., Owen A.M. (2003). Encoding strategies dissociate prefrontal activity from working memory demand. Neuron.

[6] Busch R.M., Farrell K., Lisdahl-Medina K., Krikorian R. (2005). Corsi block-tapping task performance as a function of path configuration. J. Clin. Exp. Neuropsychol..

[7] Ginsberg E.S., Rinehart N., Fielding J. (2017). Measures of task demand and error analysis in the Corsi Block-Tapping Test. Psychol. Neurosci..

[8] Orsini A., Pasquadibisceglie M., Picone L., Tortora R. (2001). Factors which influence the difficulty of the spatial path in Corsi’s block-tapping test. Percept. Mot. Ski..

[9] Orsini A., Simonetta S., Marmorato M.S. (2004). Corsi’s block-tapping test: Some characteristics of the spatial path which influence memory. Percept. Mot. Ski..

[10] Parmentier F.B., Elford G., Maybery M. (2005). Transitional information in spatial serial memory: Path characteristics affect recall performance. J. Exp. Psychol. Learn. Mem. Cogn..

[11] Smirni P., Villardita C., Zappalá G. (1983). Influence of different paths on spatial memory performance in the block-tapping test. J. Clin. Exp. Neuropsychol..

[12] Konstantinou N., Beal E., King J.R., Lavie N. (2014). Working memory load and distraction: Dissociable effects of visual maintenance and cognitive control. Atten. Percept. Psychophys..

[13] Murphy G., Groeger J.A., Greene C.M. (2016). Twenty years of load theory—Where are we now, and where should we go next?. Psychon. Bull. Rev..

[14] Burnham B.R., Sabia M., Langan C. (2014). Components of working memory and visual selective attention. J. Exp. Psychol. Hum. Percept. Perform..

[15] Konstantinou N., Lavie N. (2013). Dissociable roles of different types of working memory load in visual detection. J. Exp. Psychol. Hum. Percept. Perform..

[16] Forster S., Lavie N. (2009). Harnessing the wandering mind: The role of perceptual load. Cognition.

[17] Lavie N., Beck D.M., Konstantinou N. (2014). Blinded by the load: Attention, awareness and the role of perceptual load. Philos. Trans. R. Soc. B Biol. Sci..

[18] Lavie N. (1995). Perceptual load as a necessary condition for selective attention. J. Exp. Psychol. Hum. Percept. Perform..

[19] Lavie N. (2005). Distracted and confused?: Selective attention under load. Trends Cogn. Sci..

[20] Dalton P., Lavie N., Spence C. (2009). The role of working memory in tactile selective attention. Q. J. Exp. Psychol..

[21] Baddeley A. (2003). Working memory: Looking back and looking forward. Nat. Rev. Neurosci..

[22] Sala S.D., Baddeley A., Papagno C., Spinnler H. (1995). Dual-task paradigm: A means to examine the central executive. Ann. N. Y. Acad. Sci..

[23] Tapper A., Gonzalez D., Roy E., Niechwiej-Szwedo E. (2017). Executive function deficits in team sport athletes with a history of concussion revealed by a visual-auditory dual task paradigm. J. Sport. Sci..

[24] Kemps E. (2001). Complexity effects in visuo-spatial working memory: Implications for the role of long-term memory. Memory.

[25] Rossi-Arnaud C., Pieroni L., Baddeley A. (2006). Symmetry and binding in visuo-spatial working memory. Neuroscience.

[26] Rossi-Arnaud C., Pieroni L., Spataro P., Baddeley A. (2012). Working memory and individual differences in the encoding of vertical, horizontal and diagonal symmetry. Acta Psychol..

[27] Smyth M.M., Pendleton L.R. (1989). Working memory for movements. Q. J. Exp. Psychol..

[28] Vandierendonck A., Kemps E., Fastame M.C., Szmalec A. (2004). Working memory components of the Corsi blocks task. Br. J. Psychol..

[29] Hurlstone M.J., Hitch G.J., Baddeley A.D. (2014). Memory for serial order across domains: An overview of the literature and directions for future research. Psychol. Bull..

[30] Tapper A., Staines W.R., Niechwiej-Szwedo E. (2022). EEG reveals deficits in sensory gating and cognitive processing in asymptomatic adults with a history of concussion. Brain Inj..

[31] Bor D., Seth A.K. (2012). Consciousness and the prefrontal parietal network: Insights from attention, working memory, and chunking. Front. Psychol..

[32] Smyth M.M., Scholey K.A. (1994). Interference in immediate spatial memory. Mem. Cogn..

[33] Morra S. (2015). How do subvocal rehearsal and general attentional resources contribute to verbal short-term memory span?. Front. Psychol..

[34] Lewandowsky S., Oberauer K. (2008). The word-length effect provides no evidence for decay in short-term memory. Psychon. Bull. Rev..

[35] Ruchkin D., Grafman J., Cameron K., Berndt R. (2003). Working memory retention systems: A state of activated long-term memory. Behav. Brain Sci..

[36] Arsalidou M., Pascual-Leone J., Johnson J., Kotova T. (2019). The constructive operators of the working mind: A developmental account of mental-attentional capacity. Russ. J. Cogn. Sci..

[37] Bernstein D.M. (2002). Information processing difficulty long after self-reported concussion. J. Int. Neuropsychol. Soc..

[38] Ozen L.J., Itier R.J., Preston F.F., Fernandes M.A. (2013). Long-term working memory deficits after concussion: Electrophysiological evidence. Brain Inj..

[39] Thériault M., De Beaumont L., Tremblay S., Lassonde M., Jolicoeur P. (2011). Cumulative effects of concussions in athletes revealed by electrophysiological abnormalities on visual working memory. J. Clin. Exp. Neuropsychol..

[40] De Beaumont L., Theoret H., Mongeon D., Messier J., Leclerc S., Tremblay S., Ellemberg D., Lassonde M. (2009). Brain function decline in healthy retired athletes who sustained their last sports concussion in early adulthood. Brain.

[41] Gosselin N., Thériault M., Leclerc S., Montplaisir J., Lassonde M. (2006). Neurophysiological anomalies in symptomatic and asymptomatic concussed athletes. Neurosurgery.

[42] Witt S.T., Lovejoy D.W., Pearlson G.D., Stevens M.C. (2010). Decreased prefrontal cortex activity in mild traumatic brain injury during performance of an auditory oddball task. Brain Imaging Behav..

[43] Waters C. (2010). Investigating Selective Attention after Mild to Moderate Traumatic Brain Injury Using Perceptual Load Theory. Ph.D. Thesis.

